# Modification of the Ceramic Implant Surfaces from Zirconia by the Magnetron Sputtering of Different Calcium Phosphate Targets: A Comparative Study

**DOI:** 10.3390/ma11101949

**Published:** 2018-10-11

**Authors:** Anna I. Kozelskaya, Evgeny N. Bolbasov, Alexey S. Golovkin, Alexander I. Mishanin, Alice N. Viknianshchuk, Evgeny V. Shesterikov, Andrey Ashrafov, Vadim A. Novikov, Alexander Y. Fedotkin, Igor A. Khlusov, Sergey I. Tverdokhlebov

**Affiliations:** 1 Laboratory for Plasma Hybrid Systems, The Weinberg Research Center, School of Nuclear Science & Engineering, Tomsk Polytechnic University, 634050 Tomsk, Russia; annakozelskaya@gmail.com (A.I.K.); ebolbasov@gmail.com (E.N.B.); shesterikov_e@mail.ru (E.V.S.); aerografi@mail.ru (A.A.); fedotkin_sasha@mail.ru (A.Y.F.); 2 Institution of molecular biology and genetics, Almazov National Medical Research Centre, 197341 Saint Petersburg, Russia; golovkin_a@mail.ru (A.S.G.); mishaninssma@yandex.ru (A.I.M.); alice-vik@mail.ru (A.N.V.); 3 Laboratory of Lidar Methods, V.E. Zuev Institute of Atmospheric Optics SB RAS, 634055 Tomsk, Russia; 4 Faculty of Physics, Department of Semiconductor Physics, Tomsk State University, 634050 Tomsk, Russia; novikovvadim@mail.ru; 5 Research School of Chemistry & Applied Biomedical Sciences, Tomsk Polytechnic University, 634050 Tomsk, Russia; khlusov63@mail.ru; 6 Department of Morphology and General Pathology, Siberian State Medical University, 634050 Tomsk, Russia; 7 Institute of Living Systems, Immanuel Kant Baltic Federal University, 236041 Kaliningrad, Russia

**Keywords:** ZrO_2_, RF magnetron sputtering, surfaces, biomedical applications

## Abstract

In this study, thin calcium phosphate (Ca-P) coatings were deposited on zirconia substrates by radiofrequency (RF) magnetron sputtering using different calcium phosphate targets (calcium phosphate tribasic (CPT), hydroxyapatite (HA), calcium phosphate monobasic, calcium phosphate dibasic dehydrate (DCPD) and calcium pyrophosphate (CPP) powders). The sputtering of calcium phosphate monobasic and DCPD powders was carried out without an inert gas in the self-sustaining plasma mode. The physico-chemical, mechanical and biological properties of the coatings were investigated. Cell adhesion on the coatings was examined using mesenchymal stem cells (MSCs). The CPT coating exhibited the best cell adherence among all the samples, including the uncoated zirconia substrate. The cells were spread uniformly over the surfaces of all samples.

## 1. Introduction

In recent years, there has been a shift from amputation to treatment with endoprosthetics, which is driven by the desire to improve surgical techniques. Thus, the number of patients with surgically implanted artificial devices in bone and joint tissues is currently increasing [1,2]. These implants allow people to lead more normal lives without feeling restricted in movement. In the medical field, ceramics are used to construct prostheses or their parts to manufacture medical instruments, as membranes for the separation and purification of biological fluids, and to produce porous elements for dosed drug administration [3,4,5]. As biologically inert and electrolytically passive materials, ceramics do not conduct electric current. Ceramics also show good integration ability with bone tissue, making them advantageous in comparison with metal implants [6,7,8,9,10,11].

In addition to the above-mentioned advantages, ceramics have some significant disadvantages when used as implant materials [3]. For example, the passivity of ceramics with respect to living tissue means that the bone may not grow into the implant, and the contact site will be filled with a fibrous tissue that covers the foreign body. The high strength of ceramic materials results in considerable rigidity. This can lead to the local redistribution of load on the bone at the contact area between the implant and bone, often resulting in the destruction of the bone. In addition, as a result of the brittle nature of ceramics, the ceramic itself can be susceptible to fractures under certain conditions. Despite these disadvantages, ceramic implants have no alternatives in some areas of traumatology and orthopaedics, including the endoprostheses of small joints (finger, foot and wrist joints), ankle bone, intervertebral discs, femoral head, knee joint and teeth [12,13,14,15]. Ceramic steel made from alumina (Al_2_O_3_) or zirconia (ZrO_2_) is the most widely used ceramic implant material [16,17,18,19,20].

Biomedical-grade zirconia is usually stabilised with 3 mol% yttria (Y_2_O_3_) because yttria decreases the driving force for the tetragonal-to-monoclinic transformation. Zirconia ceramics stabilised with yttrium oxide provide several advantages over other ceramics, including excellent mechanical and tribological properties along with biocompatibility. Therefore, yttrium-oxide-stabilised zirconia ceramics are widely applied as ball heads in hip-joint implants. Furthermore, zirconia ceramics are aesthetically more appealing as dental implants compared to grey titanium implants [21,22,23,24].

The implant-bone system only functions properly when the bone tissue is fully integrated with the implant. In order to improve this integration between the bone tissue and the implant, various bioactive coatings are commonly deposited on implant surfaces [25,26]. Oxidising treatment [27,28], electrolytic deposition [29], plasma spraying [30] and vacuum-ion plasma deposition [31,32] are effective methods for producing bioinert and bioactive coatings on metal implant surfaces. Recent research efforts have focused on reducing the thickness of implant coatings to the nano level [33,34,35,36,37]. Numerous studies have shown that the micro- and nanotopography of the implant surface strongly affects the biological properties of the implant (e.g., cell attachment, spreading and proliferation) [38,39,40]. Thus, greatly reducing the film thickness can significantly change the chemistry of the implant surface without affecting its morphology [37]. The use of ultrathin coatings on dental implant materials prevents the implant from loosening, which is caused by the gap between the implant and the healthy bone in the case of rapid dissolution of the thick coating and its incomplete replacement by bone tissue [41]. In addition, thin coatings adhere much more strongly to the substrate than thicker coatings, especially in the case of screw-shaped implants.

Few studies have focused on the modification of ceramic implants. The promising methods to produce the thick ceramic coatings on ceramic substrates are electrophoretic deposition and airbrush spraying [42,43]. The available studies used various techniques for thin-film deposition. Because the deposition method significantly affects the physical, chemical and, consequently, medical and biological properties of the implant, it is difficult to determine the optimal coating for a ceramic implant and assess its clinical effectiveness. We are unaware of any studies that comparatively analysed coatings with different compositions deposited on ceramic substrates using the same deposition method.

Calcium phosphate materials, such as hydroxyapatite, tricalcium phosphate (α,β), dicalcium phosphate dihydrate and octacalcium phosphate, possess great potential as materials for bioactive coatings [26,44,45]. This paper provides a comparative study of coatings deposited by the radiofrequency (RF) magnetron sputtering of different Ca-P powders on zirconia substrates. Unlike other methods, RF magnetron sputtering allows the deposition of bioactive coatings on the surfaces of metal implants along with ceramic and polymer substrates [46,47,48]. RF-sputtered coatings provide strong adhesion to various substrates, and they also provide high elasticity, spatial uniformity and the ability to coat implants with complex shapes. Generally, coatings formed using plasma-chemical deposition methods are amorphous and can be irreversibly transformed into the crystalline state [49]. This property can be used to control the biomedical properties of the coatings.

## 2. Materials and Methods

### 2.1. Materials

Five different commercially available Ca-P powders were used in this study: calcium phosphate tribasic (H_2_Ca_10_O_26_P_6_), hydroxyapatite [Ca_10_(PO_4_)_6_(OH)_2_], calcium phosphate monobasic (H_4_CaO_8_P_2_), calcium phosphate dibasic dihydrate (HCaO_4_P·2H_2_O) and calcium pyrophosphate (Ca_2_O_7_P_2_). All powders were purchased from Sigma-Aldrich Chemie GmbH (Germany).

Partially yttrium-stabilised zirconium dioxide (ZrO_2_ + 3 mol% Y_2_O_3_) plates with diameters of 10 mm and thicknesses of 2 mm were used as the substrates. The plates were provided by MOJE Keramik-Implantate Ltd., Tomsk, Russia. In order to measure film thickness, the Ca-P coatings were sputtered onto Si plates.

Cell adhesion on the Ca-P coatings was examined using MSCs. Adipose-derived MSCs were collected from healthy donors immunophenotyped using a flow cytometer (GuavaEasyCyte6, Millipore, Burlington, MA, USA) with CD19, CD34, CD45, CD73, CD90 and CD105 monoclonal antibodies (BD, Franklin Lakes, NJ, USA), as previously described [50]. All experiments were performed according to the Declaration of Helsinki, and approval was obtained from the local ethics committees of the Almazov Federal Medical Research Centre (Saint Petersburg, Russia) (Ethical permit number 12.26/2014 from 1 December 2014). Written consent was obtained from all subjects prior to the biopsy of fat tissue. The cells were maintained in alpha-MEM medium (PanEco, Moscow, Russia) supplemented with 10% fetal calf serum (Hyclone, Logan, UT, USA), 50 units/mL penicillin and 50 µg/mL streptomycin (Invitrogen, Waltham, MA, USA) at 37 °C and 5% CO_2_.

### 2.2. Methods

#### 2.1.1. Coating Deposition

Coatings were deposited using a universal magnetron sputtering system based on the setup «Cathod 1M» in the Hybrid Materials Laboratory of Tomsk Polytechnic University [51] (Tomsk, Russia) at a generator frequency of 13.56 MHz. The distance from the target to the substrate was 38 mm, and the base pressure was 7 × 10^−3^ Pa. The pressure in the chamber was 0.3 Pa. The calcium phosphate monobasic and the DCPD powders began to release gas during sputtering, prompting us to decrease the power and cut off the flow of argon. Thus, further sputtering was performed in the self-sustaining plasma mode without any inert gas. Meanwhile, the sputtering of HA, CPT and CPP powders were carried out with argon. The operational parameters of RF magnetron sputter deposition are presented in Table 1. Prior to deposition, the ceramic substrates were prepared by grinding, followed by polishing. The discs were then ultrasonically cleaned in an ultrasonic bath (Sapfir 5, St. Petersburg, Russia) and soaked in chloroform followed by ethyl alcohol.

#### 2.1.2. Coating Investigations

*Atomic Force Microscopy*. The surface morphologies of the coatings were examined using atomic force microscopy (AFM; Solver-HV, NT-MDT, Moscow, Russia) operating in tapping mode. Root-mean-square surface roughness (Sq) was evaluated over 4 µm areas.

*Mechanical Properties*. The hardness and elastic modulus of each Ca-P coating were measured by nanoindentation test (NanoTest 600 apparatus, Wrexham, UK) using a Berkovich tip.

*Coating Thickness*. The thicknesses of the Ca-P coatings were determined using the profilometric method (Talysurf 5 profilometer-profilograph, Taylor Hobson, Leicester, UK).

*X-Ray Diffraction (XRD) Analysis*. The crystal structures of the samples were investigated using XRD (Shimadzu XRD 6000 diffractometer, Kyoto, Japan) using monochromatic CuK-alpha (1.54056 Å) radiation. The accelerating voltage and beam current were 40 kV and 30 mA, respectively. The scanning angle range, scanning step size and signal collection time were 6–55°, 0.0200° and 1.5 s, respectively.

*Elemental Analysis*. Elemental composition was evaluated using scanning electron microscopy (SEM; Quanta 200 3D, FEI Company, Hillsboro, OR, USA) equipped with energy-dispersive X-ray spectroscopy (EDX; JSM-5900LV, JEOL Ltd., Tokyo, Japan). SEM-EDX was performed under low vacuum with an accelerating voltage of 10 kV.

*Fourier Transform Infrared (FTIR) Spectroscopy*. The chemical structures of the samples were studied using attenuated total reflectance Fourier transform infrared (FTIR) spectroscopy (Nicolet 6700, Thermo Scientific, Waltham, MA, USA) in the range of 800–2000 cm^−1^ (resolution = 1 cm^−1^). 

*Coating Wettability*. The wettability of each Ca-P coating was studied using an EasyDrop device (Krüss, Hamburg, Germany) with the ‘sit’ drop method by measuring the contact angle of a 3 µL liquid drop one minute after placing it on the sample surface. To avoid surface contamination, contact angle measurements were carried out immediately after surface modification. Dimethylformamide and water were used as the wetting liquids. The total surface energy along with its polar and dispersion components was evaluated using the Owens–Wendt–Rabel–Kaelble method.

*Cell culturing* in vitro. The cell suspension was freshly prepared at a concentration of 0.5 × 10^5^ viable cells/mL in 1.5 mL of the following culture medium: 90% alpha-MEM medium (PanEco, Moscow, Russia) supplemented with 10% fetal calf serum (Hyclone, South Logan, UT, USA), 50 units/mL penicillin and 50 µg/mL streptomycin (Invitrogen, Waltham, MA, USA). A zirconium substrate with a two-sided RF-magnetron CP coating was placed in a plastic well of a 24-well flat-bottom plate (Orange Scientific, Braine-l’Alleud, Belgium). Cells were seeded on the tested samples and around them. The cell culture was incubated for 72 h at 37 °C in a humidified atmosphere of 95% air and 5% CO_2_.

*Fluorescence Microscopy of Cells*. In order to analyse the effects of surface modification on cell adhesion and attachment, focal adhesion and cytoskeletal proteins (vinculin and α-actin) were studied using fluorescence microscopy. After 72 h of incubation with cells, the samples were removed from the media, washed with phosphate-buffered saline (PBS) and fixed with 4% paraformaldehyde for 20 min. The cells were permeabilised using Triton X-100 (Sigma Aldrich, Saint Louse, MO, USA), rinsed with PBS, blocked with 10% goat serum in PBS for 30 min at room temperature and incubated with anti-vinculin antibody (diluted in PBS 1:200, Thermo Scientific, Waltham, MA, USA) for 2 h. After washing three times with PBS, the cells were incubated with Alexa Fluor 488 goat anti-mouse IgG (H + L) (diluted 1:200, Invitrogen, Waltham, MA, USA) for 1 h at room temperature in the dark. MSCs were stained with α-smooth muscle actin (α-SMA; Diagnostic BioSystems, Pleasanton, CA, USA) and Alexa Fluor 546 goat anti-mouse IgG (H + L) (Invitrogen). The cells were then washed three times with PBS (5 min each) and stained with 4′,6-diamidino-2-phenylindole (DAPI) for nuclear visualisation. After final washing, the stained cells were viewed by fluorescence microscopy (Carl Zeiss Axio Observer microscope, ZEISS, Jena, Germany). Images were collected and processed with Zen Software. Morphometric analysis was performed using ImageJ. All fluorescence microscopy analyses were performed at the Almazov National Medical Research Centre (Saint Petersburg, Russia). 

*Cell viability*. For cytotoxicity analysis by flow laser cytometry with the determination of the number of dead (apoptotic) cells. Cell culture was detached from the surface with a 1% trypsin solution in EDTA (Sigma Aldrich, St. Louis, MO, USA). The cell suspensions were stained with Annexin V FITC (Biolegend, San Diego, CA, USA) and Propidium Iodide (Sigma Aldrich), according to the manufacturer’s recommendations. The samples were analyzed on a Guava Easy Cyte 8 (Millipore, Burlington, MA, USA) flow cytometer. The results were analyzed in Kaluza (Beckman Coulter, Brea, CA, USA) software environment. 

*Statistical Analysis*. Statistical analysis was performed using Statistica 7.0 software (StatSoft, Tulsa, OK, USA). The data are presented as mean ± standard deviation. Significant differences were determined using one-way analysis of variance and Mann–Whitney U test.

## 3. Results and Discussion

**Figure 1:**
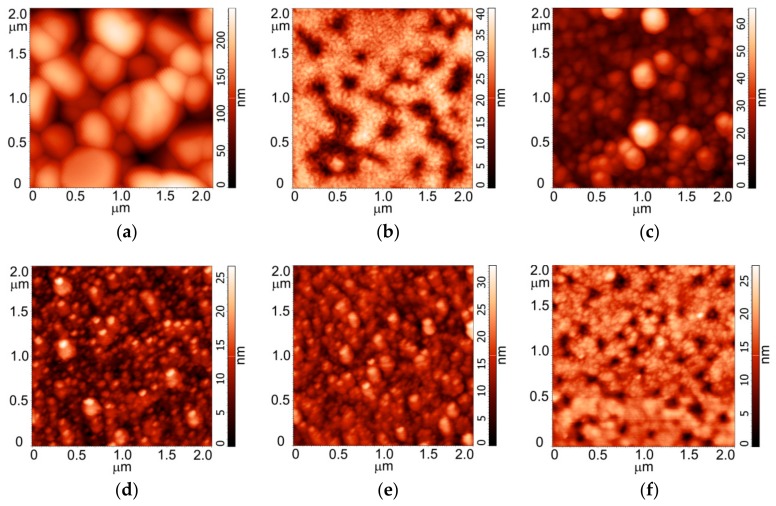
AFM images of the Ca-P coatings on the ZrO_2_ substrates: (**a**) ZrO_2_ substrate; (**b**) CPT coating; (**c**) HA coating; (**d**) Calcium phosphate monobasic coating; (**e**) DCPD coating; (**f**) CPP coating.

Based on the AFM data, the zirconia substrate was characterised by relatively equiaxed grains with an average size of 130 nm and an Sq of 30 nm (Figure 1a). Coating the zirconia substrate with Ca-P powders using RF magnetron sputtering led to changes in its morphology. The surface of the CPT coating was wavy as a result of alternating hillocks and valleys (Figure 1b). Higher magnification revealed a plate-like structure composed of thin plates with lengths of 80 nm and widths of 30 nm. Sq of the CPT coating was 7.00 nm. The surface of the HA powder coating consisted of spherical globules with a mean size of ~20 nm and an Sq of 9.10 nm (Figure 1c). The calcium phosphate monobasic, DCPD and CPP coatings had more homogeneous structures with average grain sizes of 13.50, 10.60 and 7.60 nm, respectively (Figure 1d–f) and similar Sq values of 3.60, 3.80 and 3.60 nm, respectively. 

It is necessary to consider the differences in the mechanical properties between the coating and the substrate as significant differences in mechanical properties can cause stress at the coating–substrate interface, resulting in delamination [52,53]. The mechanical properties of the Ca-P coatings in this study are presented in Table 2. 

According to the international standard ISO 14577, the nanoindentation method allows the measurement of mechanical characteristics in three size ranges: nano (h_max_ ≤ 200 nm), micro (h_max_ > 200 nm and F_max_ < 2 N) and macro (2 N ≤ F_max_ ≤ 30 kN) [54]. In this study, in order to exclude the influence of the substrate when measuring the mechanical properties of the coatings, the load on the indenter did not exceed 0.5 mN, and the maximum indenter penetration depth was 90 nm.

Based on the nanoindentation data, the nanohardness of the CPT coating was comparable to that of the zirconia substrate. In contrast, the calcium phosphate monobasic coating exhibited the lowest nanohardness and elastic modulus among all samples, and its nanohardness was significantly different from that of the substrate. The nanohardness and elastic modulus values of the HA, DCPD and CPP coatings were practically the same.

The good elastic properties of the zirconia substrate coated with CPT powder were confirmed by its higher elastic recovery value (*R*) compared to the other samples. The value of *R* depends on the modulus of elasticity of the material. In noncrystalline materials, the elastic component arises from the elastic displacement of the points of the structural grid under external load; its value is proportional to the mechanical compliance of the material.

XRD analysis indicated that all the deposited Ca-P coatings were amorphous. The elemental compositions of the coatings are presented in Table 3. Here, we primarily consider the elements Ca and P, which are important for cell activity [53] and contribute to the formation of bone tissue [55]. The largest content of Ca was observed in the HA coating, followed by the DCPD coating. The trend in P content in the Ca-P coatings was similar to the trend observed for Ca content. The largest P contents were observed in the HA and DCPD coatings. The presence of Y, Zr and O elements was attributed to the substrate composition, whereas the presence of C was associated with the deposition process. All samples are characterized by practically the same Ca/P content ratio that varies in the range 1.84–2.33. Such composition is usual for RF-sputtered Ca-P coatings on metallic substrates [56]. Highly likely, a variation of Ca/P ratio had no significant effect on diverse cell adhesion described in Table 4.

**Figure 2:**
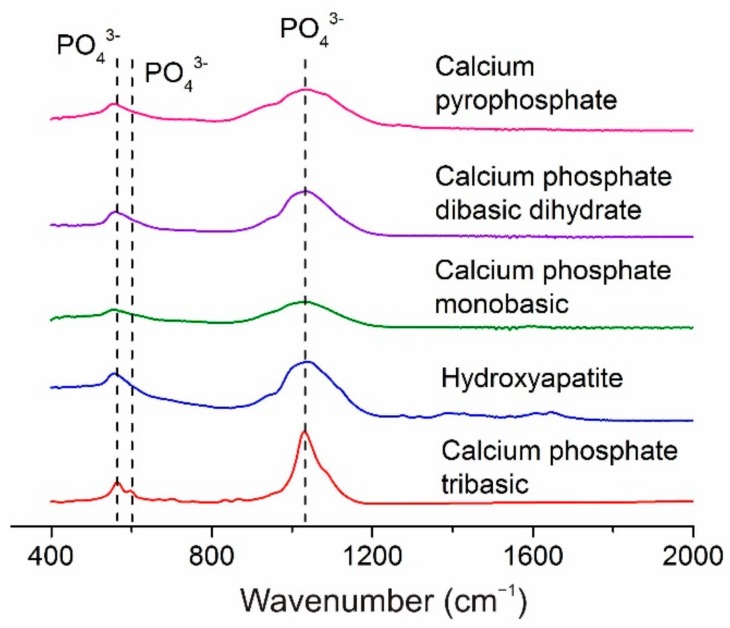
FTIR spectra of the as-deposited Ca-P coatings.

The FTIR spectra of all the investigated coatings exhibited weak absorption bands at 560 and 600 cm^−1^ along with an absorption band between 1000 and 1200 cm^−1^ (Figure 2). The intensities of the latter bands were greatest in the spectrum of the CPT coating. The bands mentioned above correspond to the valence vibrations of the PO_4_^3−^ group [57,58]. No other characteristic absorption bands were observed in the FTIR spectra of the Ca-P coatings in this study.

The deposition of Ca-P coatings on the zirconia substrates led to changes in the surface free energy (Table 5). The HA coating resulted in the largest surface free energy, whereas CPP produced the smallest value (Table 2).

The surface free energy data are in good agreement with the roughness data; that is, the greatest roughness value corresponds to the largest surface free energy. Micro-relief and surface roughness are known to be closely related to surface free surface [59] and, consequently, to surface wettability [60,61]. Compared to a smooth surface, surface roughness increases the contact area between the liquid and the solid. In turn, this results in a proportional increase in the specific surface free energy of the rough surface.

Accordingly, Fan and Lee [59] observed a linear increase in the surface free energy of WN_x_ films with increasing Sq. Barshilia et al. [62] demonstrated that substrate roughness affected the superhydrophobic behaviour of polytetrafluoroethylene thin films. The authors showed that the large water contact angle and low hysteresis of the contact angle corresponded to low surface free energy values. Moreover, they established an optimum Sq value at which the polytetrafluoroethylene films exhibited superhydrophobic properties. These results are in good agreement with the data obtained for the Ca-P coatings in this study.

The wettability results indicated that the DCPD, CPT and CPP coatings had hydrophobic properties, whereas the HA and calcium phosphate monobasic coatings exhibited strong and weak hydrophilic properties, respectively. For these coatings, the polar component of the free surface energy is maximal, which indicates a high wettability of these coatings by polar liquids.

Surface free energy and surface wettability have important effects on the interaction between the implant surface and the biological environment [62,63,64]. Numerous studies have shown that high surface free energy and good wettability (hydrophilicity) facilitate cell adhesion, whereas hydrophobic surfaces with low surface free energies are unfavorable for cell attachment and spreading [65,66,67].

However, greater part of results was received on metal [68], glass and plastic substrata [64]. Some studies have found that a contact angle of 60–70° is optimal for cell adherence and direct proliferation [65,66]; at this time, other studies have failed to confirm this conclusion [63,65,67,68]. Therefore, the wettability and surface energy (*SE*) are recognized as the fundamental physicochemical properties of a material surface, which strongly but contradictorily influences the attachment of cells [69,70,71,72].

The present article focuses on the widely exploited biomaterial Ca-P coatings prepared by magnetron sputtering a set of methods, such as sol-gel, plasma spraying, microarc oxidation, detonation gas spraying methods, etc. A wide range of cells (fibroblasts, osteoblasts, osteoclasts, nerve cells, and embryonic cells, among others) respond to artificial surfaces as described, in particular in [73]. However, the connection between the multiple extracellular physicochemical events that control and trigger MSCs is still unclear.

**Figure 3:**
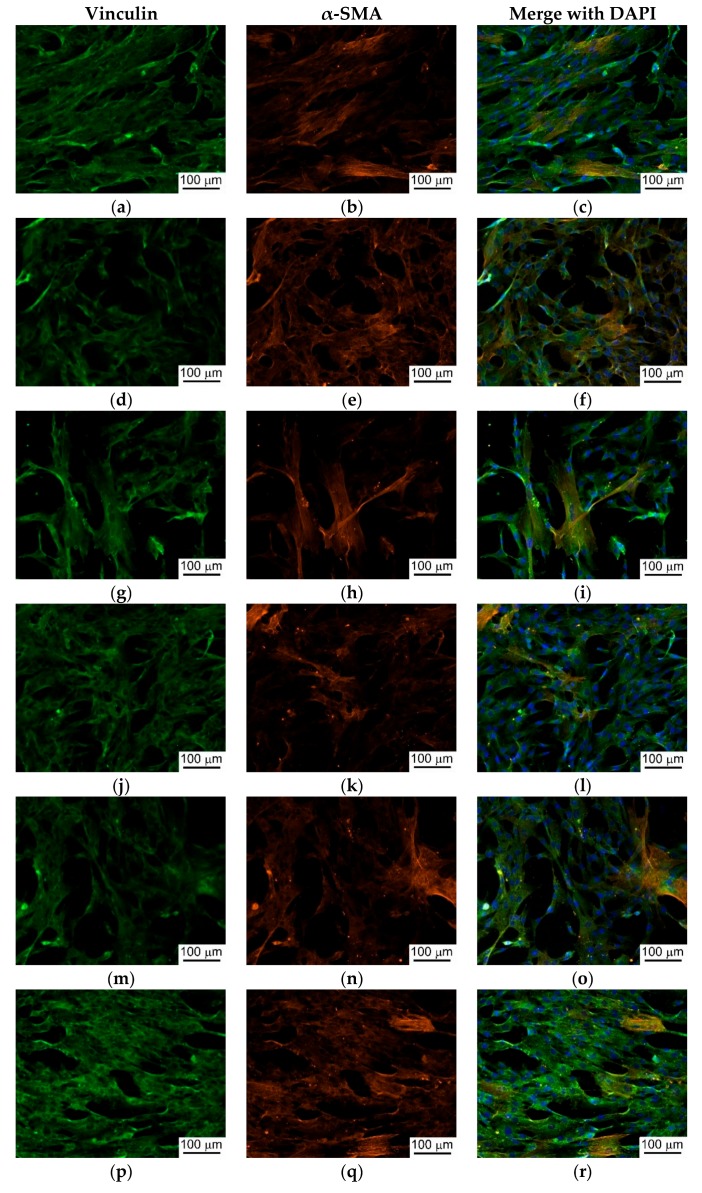
Immunofluorescence staining of MSCs cultured on ZrO_2_ substrates with different Ca-P coatings: (**a**–**c**) ZrO_2_; (**d**–**f**) CPT; (**g**–**i**) HA; (**j**–**l**) calcium phosphate monobasic; (**m**–**o**) DCPD; (**p**–**r**) CPP. Scale bar: 100 µm.

One of the main distinguishing features of MSC cultures is their ability to adhere to and spread on material surfaces; thus, the area occupied by a single cell is an important characteristic of its functional activity. Furthermore, a larger number of cells and their surface area corresponds to a greater functional activity [74,75] and biocompatibility of artificial materials tested. Table 4 presents the number of adhered MSCs, the overall cell area and the area of a single cell for the Ca-P coatings in this study. Only the sample coated with CPT showed a significantly higher cell adhesion rate, as confirmed by a higher number of cells and higher overall area covered by cells. The samples coated with other calcium phosphates demonstrated similar levels of cell adhesion, Table 4). In all samples, including the zirconia substrate, the cells were well spread uniformly over the surface, and the cell density did not differ significantly between different areas on the surface. The fluorescence microscopy indicated that the adhered MSCs formed continuous cell layers on the substrates (Figure 3). Besides the diffuse staining of the cytoplasm, the numerous focal adhesions between the cells and with the material were performed after vinculin staining. Actin was observed in the trabecular meshwork in almost all cells (Figure 3). Vinculin was predominantly found in the cytoplasm, although focal adhesion was not obvious.

It is should be noted that significantly higher adhesion of the adipose-derived MSCs was observed on the hydrophobic surface with the weak polar component of the free surface energy (coating from the CPT powder; Table 4 and Table 5). RF-magnetron coating prepared from HA target had reverse properties as compared with CPT (Table 5), and the number of adherent MSCs was minimal (Table 4). The combined effect of wettability and the positive charge on the HA surface has been known to enable differential in vitro binding of inorganic ions and organic cell adhesive proteins (e.g., fibronectin, vitronectin etc.) with different surface properties, which results in accelerated or decelerated cell adhesion on HA surface [76]. Because of uniform roughness, charge and Ca/P ratio of RF-magnetron HA coatings, a single cell-mediated effect of their wettability and surface energy may be found.

The surface chemistry of materials has an interactive influence on optimal adhesion of mammalian cells, which is critical in determining the cell viability on substrate surfaces. There is the opinion that the cells prefer to adhere to hydrophilic surfaces (see above). In turn, MSCs and adipose-derived MSCs are lipophilic cells; they transform easily into fat cells [77,78] and adhere to the hydrophobic surface of polymers [79]. Thus, our results showed that adipose-derived MSCs are capable of adhering in vitro to both types of RF-magnetron CP coating, but obviously prefer hydrophobic cites. The in vitro study showed no sign of the samples’ toxic effect on MSC culture (Table 6). In this connection, diverse MSC adhesion on CPT or HA coatings did not affect their possible cytotoxicity.

## 4. Conclusions

The physicochemical, mechanical and biological properties of Ca-P coatings deposited on zirconia substrates using the RF magnetron sputtering of CPT, HA, calcium phosphate monobasic, DCPD and CPP powders were comparatively analyzed. Calcium phosphate monobasic and DCPD powders were deposited on zirconia substrates without any inert gas in the self-sustaining plasma mode because gas was released during sputtering. In contrast, CPT, HA and CPP powders were sputtered under the flow of argon. The HA target had the highest sputtering rate, whereas the calcium phosphate monobasic and DCPD targets had the lowest. 

The CPT coating exhibited a larger number of adhered cells along with a higher overall cell area compared to the zirconia substrate. In all samples, including the zirconia substrate, the cells were spread uniformly over the substrate’s surface.

This work represents the first in vitro evaluation of coatings deposited on zirconia substrates by the RF magnetron sputtering of different Ca-P powders. An analysis of cell viability, the number of adherent MSCs, and the mechanical properties of the coatings under study revealed that the CPT-derived RF-magnetron CP coating (as compared with HA target-derived ones) exhibited the best results. In order to assess the applicability and clinical effectiveness of CP coatings on ceramic implants, the ability of the coatings to stimulate bone formation should be assessed in vivo.

## Figures and Tables

**Table 1:** Operational parameters employed during RF magnetron sputter deposition.

Parameter	Calcium Phosphate Tribasic (H_2_Ca_10_O_26_P_6_)	Hydroxyapatite [Ca_10_(PO_4_)_6_(OH)_2_]	Calcium Phosphate Monobasic (H_4_CaO_8_P_2_)	Calcium Phosphate Dibasic Dehydrate (HCaO_4_P·2H_2_O)	Calcium Pyrophosphate (Ca_2_O_7_P_2_)
Power (W)	1500	1500	1000	1100	1500
RF power density (W/cm^2^)	4.6	4.6	2.5	2.9	4.6
Deposition time (h)	7	7	14	7	7
Coating thickness (nm)	100	150	100	80	100
Sputtering rate (mm/min)	2.38 × 10^−7^	3.57 × 10^−7^	1.19 × 10^−7^	1.90 × 10^−7^	2.38 × 10^−7^

**Table 2:** Mechanical properties of the Ca-P coatings ^1^.

Powder Target	P_max_, mN	h_max_, nm	*H*, GPa	E*, GPa	*R*
Calcium phosphate tribasic (CPT)	0.5	38	7.33 ± 2.59	115 ± 25	0.44
Hydroxyapatite (HA)		59	3.44 ± 0.4	78 ± 10	0.25
Calcium phosphate monobasic		91	1.67 ± 0.31	68 ± 9	0.12
Calcium phosphate dibasic dehydrate (DCPD)		63	3.10 ± 1.19	99 ± 26	0.17
Calcium pyrophosphate (CPP)		64	3.15 ± 1.13	83 ± 23	0.21
ZrO_2_ substrate		34	8.88 ± 2.13	127 ± 23	0.54

^1^ P_max_, maximum load applied to the indenter; h*_max_*, maximum penetration depth of the indenter into the coating; *H*, nanohardness; E*, modulus of elasticity; and *R*, print elastic recovery value.

**Table 3:** Elemental compositions of the Ca-P coatings (means ± standard deviations).

Powder Target	C, at.%	O, at.%	Y, at.%	P, at.%	Zr, at.%	Ca, at.%	Ca/P
Calcium phosphate tribasic (CPT)	17.17 ± 1.76	31.95 ± 0.8	1.37 ± 0.22	5.97 ± 0.55	29.65 ± 1.06	13.79 ± 0.46	2.33 ± 0.28
Hydroxyapatite (HA)	12.63 ± 0.21	31.42 ± 0.49	1.04 ± 0.14	11.11 ± 0.33	17.21 ± 0.44	26.58 ± 0.42	2.39 ± 0.11
Calcium phosphate monobasic	19.52 ± 0.94	30.77 ± 0.47	1.65 ± 0.08	5.87 ± 1.19	31.50 ± 1.13	10.69 ± 0.45	1.88 ± 0.41
Calcium phosphate dibasic dehydrate (DCPD)	15.75 ± 0.33	30.58 ± 0.24	1.39 ± 0.49	8.25 ± 0.72	27.95 ± 0.37	16.08 ± 0.57	1.96 ± 0.23
Calcium pyrophosphate (CPP)	14.81 ± 1.09	32.44 ± 0.23	1.6 ± 0.17	6.69 ± 0.82	32.26 ± 0.99	12.2 ± 0.6	1.84 ± 0.16
ZrO_2_ substrate	19.62 ± 2.43	27.71 ± 0.97	4.95 ± 0.13	-	47.71 ± 1.69	-	-

**Table 4:** Number of adhered MSCs, overall cell area and single cell area on the Ca-P coatings (means ± standard deviations). Vinculin immunostaining.

Powder Target	Number of Cells per mm^2^	Overall Cell area, % of Total Surface	Single Cell Area, µm^2^
Calcium phosphate tribasic (CPT)	284.33 ± 84.53 *^,#^	77.73 ± 8.64 *^,#^	897 ± 163
Hydroxyapatite (HA)	143.33 ± 66.66	53.91 ± 21.01	1223 ± 214
Calcium phosphate monobasic	210.00 ± 105.22	61.18 ± 17.36	1021 ± 279
Calcium phosphate dibasic dehydrate (DCPD)	190.25 ± 45.55	58.05 ± 12.31	969 ± 114
Calcium pyrophosphate (CPP)	212.18 ± 69.39	64.55 ± 12.34	1015 ± 174
ZrO_2_ substrate	185.25 ± 79.68	54.40 ± 25.20	970 ± 403

* *p* < 0.05 comparing to the ZrO_2_ substrate. ^#^ *p* < 0.05 comparing to the CP coating prepared from HA target.

**Table 5:** Contact angles and surface free energies of Ca-P coatings (means ± standard deviations).

Powder Target	Contact Angle of Water Θ, °	Contact Angle of Dymethyl Formamide Θ, °	Surface Free Energy γ, mJ/m^2^	Dispersion Component γ^d^, mJ/m^2^	Polar Component γ^p^, mJ/m^2^
Calcium phosphate tribasic (CPT)	98.0 ± 1.25	31.7 ± 4.31	38.58 ± 1.48	38.47 ± 1.43	0.11 ± 0.05
Hydroxyapatite (HA)	25.3 ± 6.16	7.8 ± 2.93	70.62 ± 2.41	6.98 ± 0.51	63.65 ± 1.90
Calcium phosphate monobasic	76.1 ± 3.89	39.8 ± 6.66	30.45 ± 2.49	17.65 ± 1.52	12.79 ± 0.96
Calcium phosphate dibasic dehydrate (DCPD)	103.0 ± 4.74	56.7 ± 3.99	25.11 ± 1.29	24.62 ± 1.14	0.50 ± 0.15
Calcium pyrophosphate (CPP)	95.7 ± 3.75	57.2 ± 2.20	22.35 ± 0.86	19.45 ± 0.60	2.90 ± 0.26
ZrO_2_ substrate	98.4 ± 2.98	53.8 ± 2.44	25.01 ± 0.84	23.64 ± 0.69	1.37 ± 0.16

**Table 6:** The percentage of dead adipose-derived MSCs after cultivation with Ca-P coatings prepared from different targets by RF-magnetron sputtering.

Powder Target	Calcium Phosphate Tribasic (CPT)	Hydroxyapatite (HA)	Calcium Phosphate Monobasic	Calcium Phosphate Dibasic Dehydrate (DCPD)	Calcium Pyrophosphate (CPP)	ZrO_2_ Substrate	Control
Dead cells, %	2.20 ± 0.62	3.37 ± 2.44	2.60 ± 1.76	3.49 ± 1.33	1.84 ± 0.69	1.45 ± 0.58	2.01 ± 0.52
